# A20’s linear ubiquitin–binding motif restrains pathogenic activation of Th17 cells and IL-22–driven enteritis

**DOI:** 10.1172/JCI187499

**Published:** 2025-09-02

**Authors:** Christopher J. Bowman, Dorothea M. Stibor, Xiaofei Sun, Nika Lenci, Hiromichi Shimizu, Emily F. Yamashita, Rommel Advincula, Min Cheol Kim, Jessie A. Turnbaugh, Yang Sun, Bahram Razani, Peter J. Turnbaugh, Chun Jimmie Ye, Barbara A. Malynn, Averil Ma

**Affiliations:** 1 Department of Medicine,; 2 Department of Pathology,; 3 Department of Epidemiology and Biostatistics,; 4 Department of Microbiology and Immunology, and; 5 Department of Dermatology, University of California, San Francisco (UCSF), San Francisco, California, USA.; 6 Gladstone-UCSF Institute of Genomic Immunology, San Francisco, California, USA.

**Keywords:** Gastroenterology, Immunology, Adaptive immunity, Inflammatory bowel disease

## Abstract

A20, encoded by the *TNFAIP3* gene, is a protein linked to Crohn’s disease and celiac disease in humans. We now find that mice expressing point mutations in A20’s M1-ubiquitin–binding zinc finger 7 (ZF7) motif spontaneously develop proximal enteritis that requires both luminal microbes and T cells. Cellular and transcriptomic profiling reveals expansion of Th17 cells and exuberant expression of IL-17A and IL-22 in intestinal lamina propria of *A20^ZF7^* mice. While deletion of IL-17A from *A20^ZF7/ZF7^* mice exacerbates enteritis, deletion of IL-22 abrogates intestinal epithelial cell hyperproliferation, barrier dysfunction, and alarmin expression. Colonization of adult germ-free mice with microbiota from adult WT specific pathogen–free mice drives duodenal IL-22 expression and duodenitis. *A20^ZF7/ZF7^* Th17 cells autonomously express more RORγt and IL-22 after differentiation in vitro. ATAC sequencing identified an enhancer region upstream of the *Il22* gene, and this enhancer demonstrated increased activating histone acetylation coupled with exaggerated *Il22* transcription in *A20^ZF7/ZF7^* T cells. Acute inhibition of RORγt normalized histone acetylation at this enhancer. Finally, CRISPR/Cas9–mediated ablation of *A20^ZF7^* in human T cells increases RORγt expression and *IL22* transcription. These studies link A20’s M1-ubiquitin binding function with RORγt expression, expansion of Th17 cells, and epigenetic activation of IL-22–driven enteritis.

## Abstract 2

Crohn’s Disease, Celiac Disease, and Intestinal Behcet’s Disease are three diseases that can afflict the proximate small bowel. The A20/TNFAIP3 gene is genetically linked to all three. We now find that mice bearing a targeted mutation of A20’s ubiquitin binding motif spontaneously develop proximate enteritis. We unveil a novel pathophysiological pathway from A20’s ZF7 motif to RORg expression, regulation of a distal IL-22 enhancer in TH17 cells, and IL-22-and-microbe driven enteritis.

## Introduction

Intestinal immune homeostasis involves complex interactions between immune and non-immune cells. Many of these interactions are mediated by cytokines, and disruption of this cytokine network can lead to intestinal disease. Type 3 cytokines such as IL-17A, IL-17F, and IL-22 support antifungal and antibacterial responses (1). These mediators can also support epithelial homeostasis and regeneration (2). Dysregulated expression of type 3 cytokines is a feature of intestinal inflammation, and expansion of Th17 cells has been consistently observed in experimental models and human inflammatory bowel disease (IBD) patients (3, 4). However, IL-17A appears to play more complex roles in the intestinal milieu than in other tissues (5, 6). Some of this complexity may be related to the ability of Th17 cells to produce other cytokines under distinct physiological conditions. Hence, understanding the physiological regulation of intestinal cytokines and cytokine responses is crucial for dissecting the adaptive versus pathological functions of these cytokine mediators.

A20/TNFAIP3 is genetically linked to IBD and celiac disease via GWAS (7–9). In addition, rare patients harboring haploinsufficient mutations of the A20 gene develop early-onset IBD as well as Behçet’s disease with intestinal ulcerations (10–12). Hence, deficiencies of A20 expression and/or function likely compromise human intestinal homeostasis. Mechanistic studies have revealed that the A20 protein regulates several signaling cascades, including TNFR-, TLR-, TCR-, NOD2-, and CD40R-triggered signals (13–19). A20 performs these functions by regulating the ubiquitination of critical signaling molecules such as RIP1, RIP2, pro–IL-1β, and RIP3 as well as ubiquitinated signaling complexes such as the IKKγ complex. Yet the mechanisms by which A20 preserves intestinal immunity are incompletely understood. This is partly because the A20 protein harbors distinct biochemical domains that mediate deubiquitinating (DUB), E3 ubiquitin (Ub) ligase, and non-catalytic linear (M1)-Ub chain binding activities (13, 14, 20–25). In this study, we unveil a unique role for A20’s M1-Ub–binding motif in restraining epigenetic regulation of IL-22 expression in Th17 cells, intestinal epithelial cell homeostasis, and microbe-dependent enteritis.

## Results

### Linear (M1)-ubiquitin–binding motif of A20 prevents T cell–dependent enteritis.

**Figure 1:**
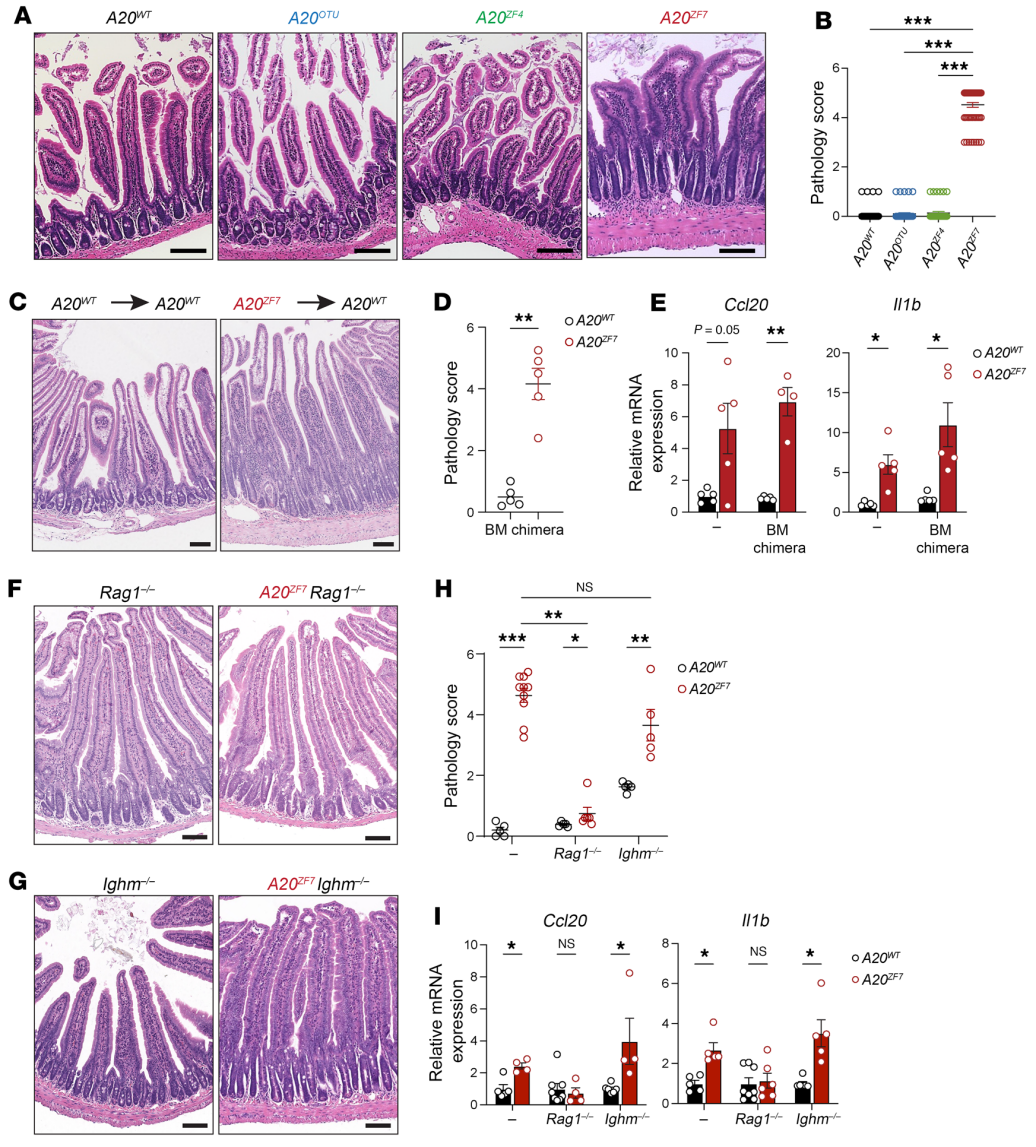
*A20^ZF7^* restrains small intestinal enteritis that is T cell dependent. (**A** and **B**) Representative H&E histology (**A**) and histopathological scores (**B**) of inflammation of proximal small intestines from indicated genotypes of mice. Scale bars: 200 μm. (**C** and **D**) Representative H&E histology (**C**) and histopathological scores (**D**) of inflammation of small intestine from radiation bone marrow chimeric mice reconstituted with indicated bone marrow genotypes. Scale bars: 100 μm. (**E**) Quantitative PCR (qPCR) analyses of indicated proinflammatory genes in proximal small intestines from indicated bone marrow chimeras. (**F**) Representative H&E histology of small intestine from indicated genotypes of *Rag1^–/–^* mice. Scale bars: 100 μm. Data are representative of at least 4 independent pairs of mice. (**G**) Representative H&E histology of small intestine from indicated genotypes of *Ighm^–/–^* mice. Scale bars: 100 μm. Data are representative of at least 3 independent pairs of mice. (**H**) Pathology scores of small intestinal inflammation. (**I**) qPCR analyses of indicated proinflammatory genes in proximal small intestines from indicated genotypes of mice. In panels above, each circle represents 1 mouse. Data are shown as mean ± SEM. Statistics were calculated using non-parametric Kruskal-Wallis test with Dunn’s multiple-comparison correction (**B**), unpaired 2-tailed Mann-Whitney *U* test (**D** and **H**), or unpaired 2-tailed Student’s *t* test with Welch’s correction (**E** and **I**). *P < 0.05, **P < 0.01, ***P < 0.001.

To understand the biochemical functions of A20 that regulate intestinal homeostasis, we analyzed intestines from a series of A20 knock-in mice that abrogate A20’s DUB (*A20^OTU^*), E3 Ub ligase (*A20^ZF4^*), or M1-Ub binding (*A20^ZF7^*) activities (20, 21). Macroscopically, small intestines from 12-week-old *A20^ZF7/ZF7^* mice, but not other genotypes, demonstrated visible thickening of the intestinal wall. Histology suggested that the proximal small intestinal mucosa of *A20^ZF7/ZF7^* mice harbored increased numbers of immune cells in comparison with congenic *A20^ZF4/ZF4^*, *A20^OTU/OTU^*, or wild-type (WT) mice (Figure 1, A and B). The expansion of the small intestinal lamina propria (SILP) by lymphocytes is reminiscent of chronic enteritis; however, other features of chronicity (e.g., epithelial metaplasia, villus blunting) were absent. This phenotype was evident in both male and female *A20^ZF7/ZF7^* mice and was 100% penetrant by 12 weeks of age. This phenotype was not evident in more distal portions of the small intestine and large intestines from 12-week-old *A20^ZF7/ZF7^* mice. In particular, ileal and colonic tissues from *A20^ZF7/ZF7^* mice expressed normal histology without molecular markers of inflammation (Supplemental Figure 1, A and B; supplemental material available online with this article; https://doi.org/10.1172/JCI187499DS1). The selective enhanced inflammation of proximal small intestines of *A20^ZF7/ZF7^* mice differs from other spontaneous models of intestinal inflammation such as *Il2^–/–^*, *Il10^–/–^*, and *Tnf^ΔARE^* mice. Hence, A20’s M1-Ub binding activity via its zinc finger 7 (ZF7) domain preserves small intestinal immune homeostasis independently of A20’s DUB and E3 Ub ligase activities.

A20 is expressed and induced in many cell types, including both hematopoietic and non-hematopoietic cells (26). To determine the degree to which hematopoietic cells of *A20^ZF7/ZF7^* mice (hereafter designated *A20^ZF7^* mice) are sufficient to drive this pathology, we generated radiation chimeras using either *A20^ZF7^* or WT bone marrow cells to reconstitute irradiated WT mice Chimeras bearing *A20^ZF7^* hematopoietic cells spontaneously developed enteritis by 12 weeks after reconstitution, a phenotype that was not seen in chimeras containing WT hematopoietic cells (Figure 1, C and D). Furthermore, the *A20^ZF7^* hematopoietic cells drove exaggerated expression of proinflammatory myeloid cytokines such as *Il1b* and chemokines such as *Ccl20* (Figure 1E). To assess the relative contributions of T and B cells to enteritis in *A20^ZF7^* mice, we interbred these mice with *Rag1**^–/–^*** and *Ighm**^–/–^*** mice. Twelve-week-old *A20^ZF7^* *Rag1**^–/–^*** mice exhibited negligible intestinal inflammation and expressed levels of *Il1b* and *Ccl20* similar to those in *Rag1**^–/–^*** mice (Figure 1, F, H, and I), suggesting that adaptive lymphocytes are required for enteritis in *A20^ZF7^* mice. By contrast, intestines from *A20^ZF7^* *Ighm**^–/–^*** mice accumulated similar numbers of lamina propria immune cells (Figure 1, G and H) and expressed similarly elevated levels of inflammatory markers in comparison with *A20^ZF7^* mice (Figure 1I). Hence, *Rag1*-dependent T lymphocytes but not B lymphocytes are required for small intestinal inflammation in *A20^ZF7^* mice.

### Type 3 cytokines and Th17 cells are increased in intestinal lamina propria of A20^ZF7^ mice.

**Figure 2:**
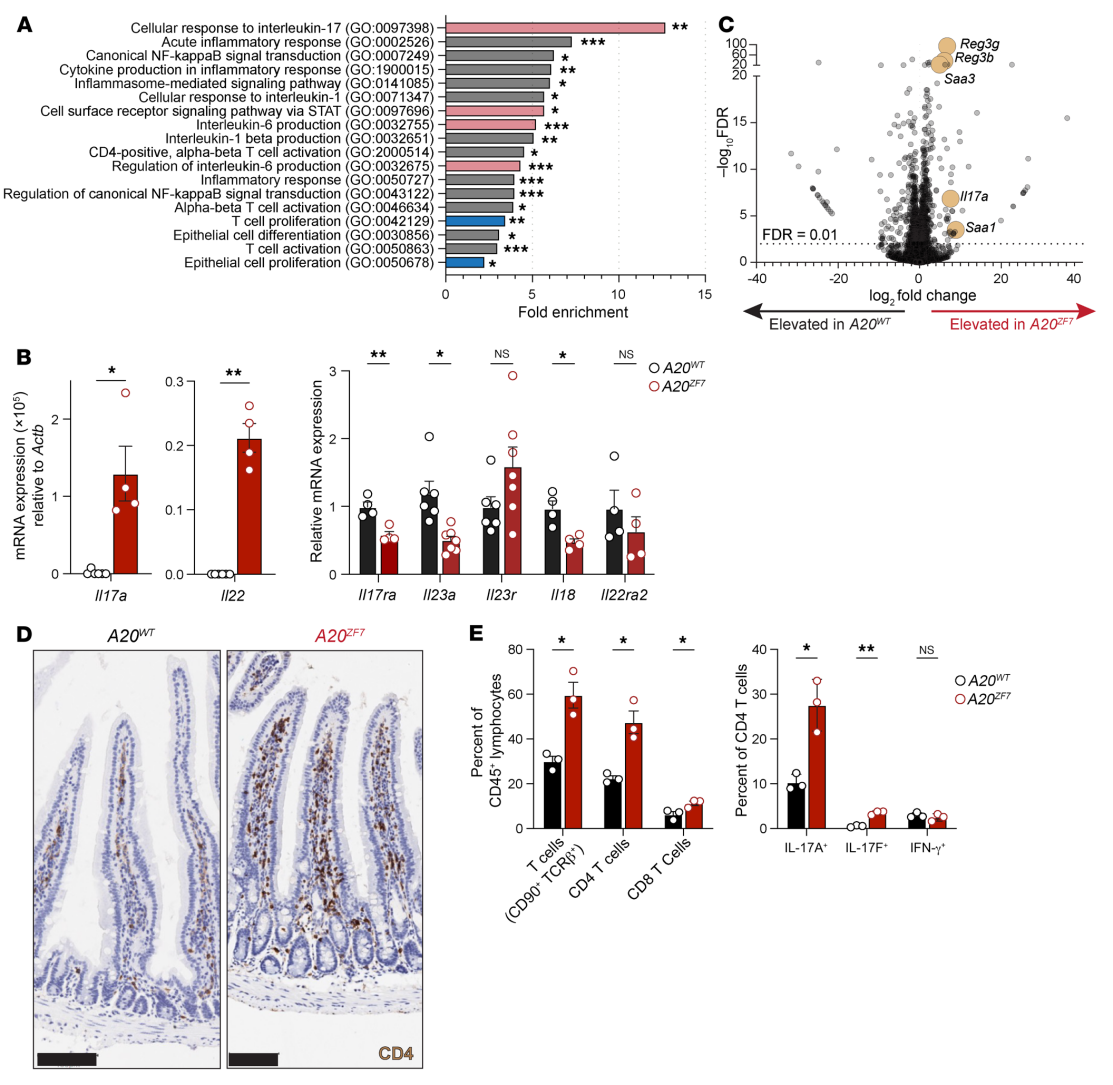
Small intestines of *A20^ZF7^* mice harbor expanded Th17 cells. (**A**) Gene ontology enrichment of genes that are significantly upregulated in *A20^ZF7^* versus WT intestines by bulk RNA-Seq. Red bars highlight categories related to Th17 differentiation. Blue bars highlight categories related to cellular proliferation. (**B**) qPCR analyses of mRNA expression from intact small intestine. (**C**) Volcano plot of all annotated UCSC RefSeq genes from bulk RNA-Seq analyses of *A20^ZF7^* versus WT small intestines. Horizontal dotted line indicates adjusted *P* value (FDR) of 0.01. (**D**) Representative immunohistochemical analyses of CD4 expression in WT and *A20^ZF7^* small intestines. Data are representative of 3 mice from each genotype. Scale bars: 100 μm. (**E**) Flow cytometry of small intestinal lamina propria (SILP) cells from WT and *A20^ZF7^* mice. SILP yields from the proximal 10 cm of small intestine typically range from 1 million to 2 million cells from WT mice and 3 million to 7 million cells from *A20^ZF7^* mice. Data are shown as mean ± SEM. Statistics were calculated using unpaired 2-tailed Student’s *t* test with Welch’s correction. *P < 0.05, **P < 0.01, ***P < 0.001.

To better understand why small intestinal inflammation develops in *A20^ZF7^* mice, we analyzed the transcriptomes of intact small intestines from 12-week-old WT and *A20^ZF7^* mice by bulk RNA sequencing (RNA-Seq). These studies revealed that *A20^ZF7^* mice upregulated genes involved in NF-κB and inflammasome signaling pathways (Figure 2A). In addition, *A20^ZF7^* intestines expressed elevated levels of genes associated with CD4^+^ T cell activation, T cell proliferation, and IL-1β production (Figure 2A). IL-17 response genes were among the most enriched groups of genes, which — together with STAT and IL-6 regulation — suggests a prominent type 3 cytokine tone in *A20^ZF7^* intestines. Indeed, quantitative mRNA analyses of intact intestines confirmed the exaggerated expression of type 3 cytokines, *Il17a* and *Il22* (Figure 2B). Meanwhile, *Il17ra* levels were decreased in *A20^ZF7^* intestines, an expected downregulation in response to elevated IL-17 (27). Surprisingly, expression of proinflammatory *Il23a* and *Il18* was diminished and *Il23r* was not significantly elevated in *A20^ZF7^* intestines (Figure 2B). IL-22–dependent transcripts, such as *Reg3b*, *Reg3g*, *Saa1*, and *Saa3*, were significantly upregulated in *A20^ZF7^* intestines (Figure 2C and Figure 5E), while expression of *Il22ra2* was not significantly altered (Figure 2B). As these data suggest that Th17 cells and/or group 3 innate lymphoid cells (ILC3s) might be expanded or hyperfunctional in *A20^ZF7^* intestines, we profiled cellular infiltrates from small intestinal tissues. Immunohistochemistry of intact small intestines indicated that CD4^+^ T cells were expanded in the lamina propria of *A20^ZF7^* mice (Figure 2D), and flow cytometry of dissociated SILP cells confirmed a relative expansion of CD4^+^ T cells (Figure 2E). The increased number of CD4^+^ T cells in *A20^ZF7^* SILP largely comprised an expansion of Th17 cells in comparison with WT littermates (Figure 2E). In addition, among expanded *A20^ZF7^* SILP CD4^+^ T cells, IL-17A– and IL-17F–expressing cells were disproportionally increased while IFN-γ–expressing cells were present in similar proportions to those of WT mice.

**Figure 3:**
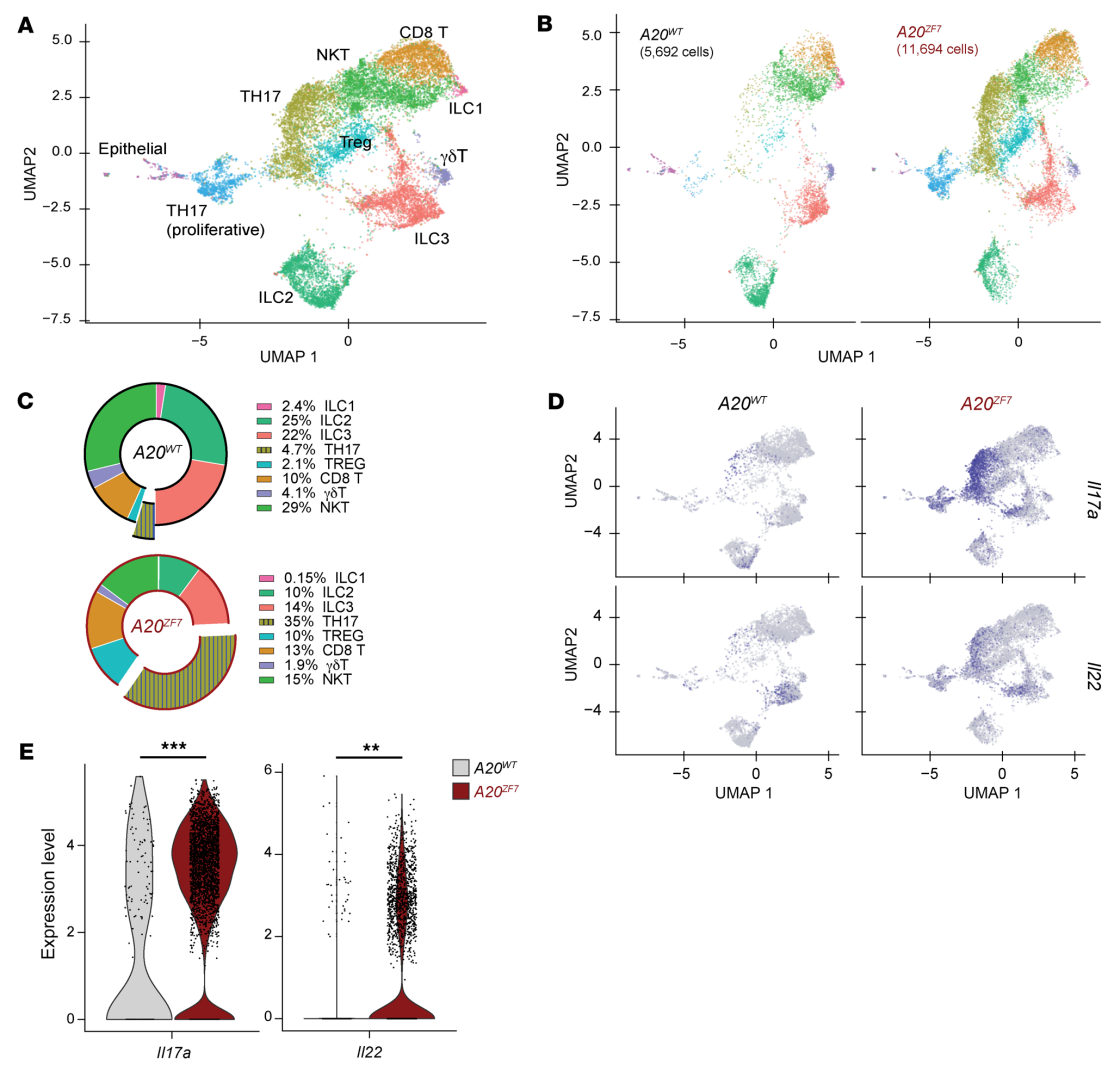
*A20^ZF7^* mice show proliferation and expansion of Th17 and regulatory T cells within the SILP. (**A** and **B**) UMAP clusters of scRNA-Seq analyses of SILP from WT and *A20^ZF7^* mice. Data represent pooled mixtures of 2 WT and 2 *A20^ZF7^* mice. (**C**) Relative proportions of lymphoid subsets in WT and *A20^ZF7^* SILP. Th17 subset includes both proliferative (mustard yellow) and non-proliferative (sky blue) compartments. (**D**) Projection of *Il17a* and *Il22* expression onto UMAP clusters shown in **B**. (**E**) Violin plots of *Il17a* and *Il22* expression in Th17 cells from indicated genotypes of mice. Statistics were calculated using unpaired 2-tailed Wilcoxon’s rank sum test. **P < 0.01, ***P < 0.001.

To better define the SILP lymphocytes in *A20^ZF7^* mice, we enriched SILP T cells from 12-week-old WT and *A20^ZF7^* mice and profiled these cells by single-cell RNA-Seq (scRNA-Seq). To avoid clustering cells based on cell cycle phases, genes related to cell cycling were removed before clustering analyses. Uniform manifold approximation and projection (UMAP) analyses of these cell cycle–regressed cells identified CD8^+^, Th17, and regulatory T cells, as well as subsets of innate lymphoid cells (ILCs) (Figure 3A and Supplemental Figure 2A). Genotype-specific UMAP analyses showed a relative expansion of CD4^+^ and, to a lesser extent, CD8^+^ T cells in *A20^ZF7^* mice (Figure 3, B and C).

Expanded clusters of CD4^+^ T cells in *A20^ZF7^* intestines contained many cells expressing *Il17a* and *Il22*, delineating these cells as Th17 cells (Figure 3D and Supplemental Figure 2A). Another expanded cluster included regulatory T cells (Figure 3C). By contrast, ILC3s were relatively reduced in *A20^ZF7^* mice (Figure 3C). In addition to being more abundant in *A20^ZF7^* mice, *A20^ZF7^* Th17 cells expressed higher levels of *Il17a* and *Il22* than WT Th17 cells (Figure 3E). The marked expansion of Th17 cells, the increased expression of type 3 cytokines, and the absence of ILC3 expansion in *A20^ZF7^* intestines relative to WT intestines suggest that Th17 cells account for the great majority of the increased type 3 cytokine production in *A20^ZF7^* intestines.

The Th17 cells segregated into 2 discrete clusters in UMAP space, and both clusters were heavily represented in *A20^ZF7^* intestines (Figure 3, A and B, and Supplemental Figure 2A). After the contributions of cell cycle genes had been removed as an effect on cell clustering, these two clusters continued to exhibit differential cell cycle states: cells with high G_0_/G_1_ scores (indicating a predominantly non-proliferative state) and cells enriched for high G_2_/M or S scores (suggesting T cell proliferation) (Supplemental Figure 2B). In addition, this “Th17 proliferative” cluster expressed high levels of *Mki67*, the gene that encodes the proliferative marker Ki67 (Supplemental Figure 2A). The accumulation of proliferative Th17 cells in *A20^ZF7^* SILP aligns with our bulk RNA-Seq analysis, which highlighted T cell proliferation as an enriched category in *A20^ZF7^* intestines (Figure 2A). Although the proliferative cluster was transcriptionally distinct from non-proliferating T cells in either *A20^ZF7^* or WT mice, it consisted of cells that expressed detectable levels of *Il17a* and/or *Il22*, confirming they were Th17 cells (Figure 3D and Supplemental Figure 2A). Hence, increased numbers of proliferating Th17 cells help explain why Th17 cells are more numerous in *A20^ZF7^* intestines.

### IL-22, but not IL-17A, drives enteritis in A20^ZF7^ mice.

**Figure 4:**
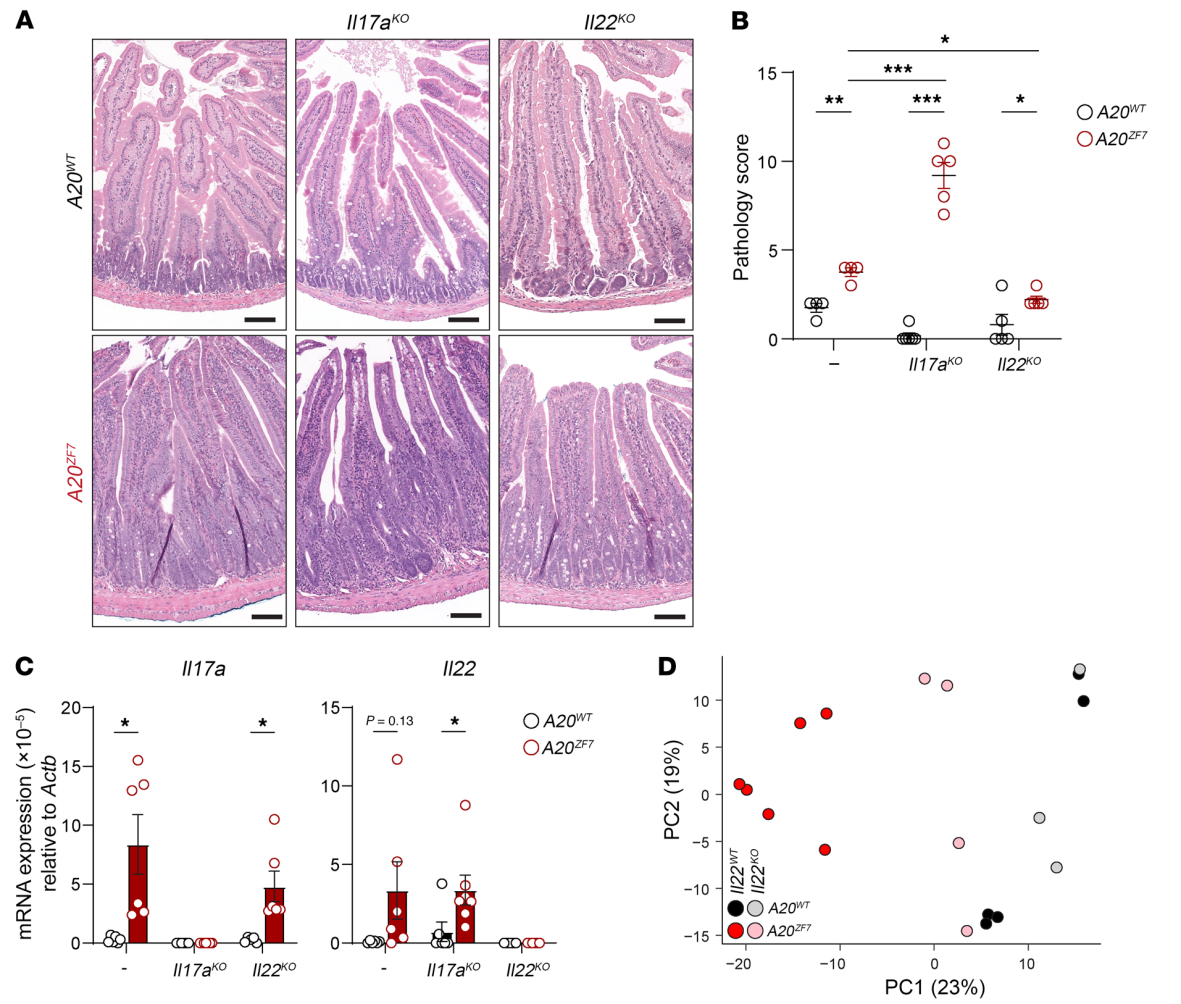
IL-17A protects against and IL-22 promotes small intestinal enteritis in *A20^ZF7^* mice. (**A** and **B**) Representative H&E histology (**A**) and pathology scores (**B**) of small intestines from mice of the indicated genotypes. Scale bars: 100 μm. (**C**) qPCR analyses of type 3 cytokines in small intestines from indicated genotypes of mice. (**D**) Principal component analyses of bulk RNA-Seq analyses of indicated genotypes of mice. Data are shown as mean ± SEM. Statistics were calculated using 2-way ANOVA with Holm-Šidák multiple-comparison correction (**B**) or unpaired 2-tailed Student’s *t* test with Welch’s correction (**C**). *P < 0.05, **P < 0.01, ***P < 0.001.

As *A20^ZF7^* mouse small intestines contain dramatic expansions of SILP Th17 cells and increased tissue-wide expression of IL-17A and IL-22, we next sought to functionally define the potential roles of these cytokines in regulating intestinal disease in *A20^ZF7^* mice. We interbred *A20^ZF7^* mice with *Il17a^–/–^* mice and *Il22^–/–^* mice. Small intestines from *A20^ZF7^* *Il17a^–/–^* mice exhibited more (rather than less) severe enteritis than those from IL-17A–competent *A20^ZF7^* mice (Figure 4, A and B). Hence, IL-17A plays a protective rather than proinflammatory role in enteritis in *A20^ZF7^* mice. *A20^ZF7^* *Il17a^–/–^* intestines expressed more *Il22* than *Il17a^–/–^* intestines but not *A20^ZF7^* intestines (Figure 4C). In contrast to *A20^ZF7^* *Il17a^–/–^* mice, *A20^ZF7^* *Il22^–/–^* mice exhibited less severe enteritis than *A20^ZF7^* mice (Figure 4, A and B). Therefore, IL-22 promotes small intestinal inflammation in *A20^ZF7^* mice. To better define the role of IL-22 in *A20^ZF7^* intestines, we profiled the genome-wide transcriptomes of small intestines from *A20^ZF7^* *Il22^–/–^* and control mice by bulk RNA-Seq. Principal component analysis of bulk RNA-Seq data revealed broad normalization toward wild-type transcriptomic states in *A20^ZF7^* *Il22^–/–^* mice when compared with *A20^ZF7^* mice (Figure 4D). Thus, IL-22 drives transcriptome-wide changes in the proximal small intestine that promote intestinal inflammation in *A20^ZF7^* mice.

### A20^ZF7^ mice exhibit IL-22– and microbe-dependent epithelial barrier dysfunction.

**Figure 5:**
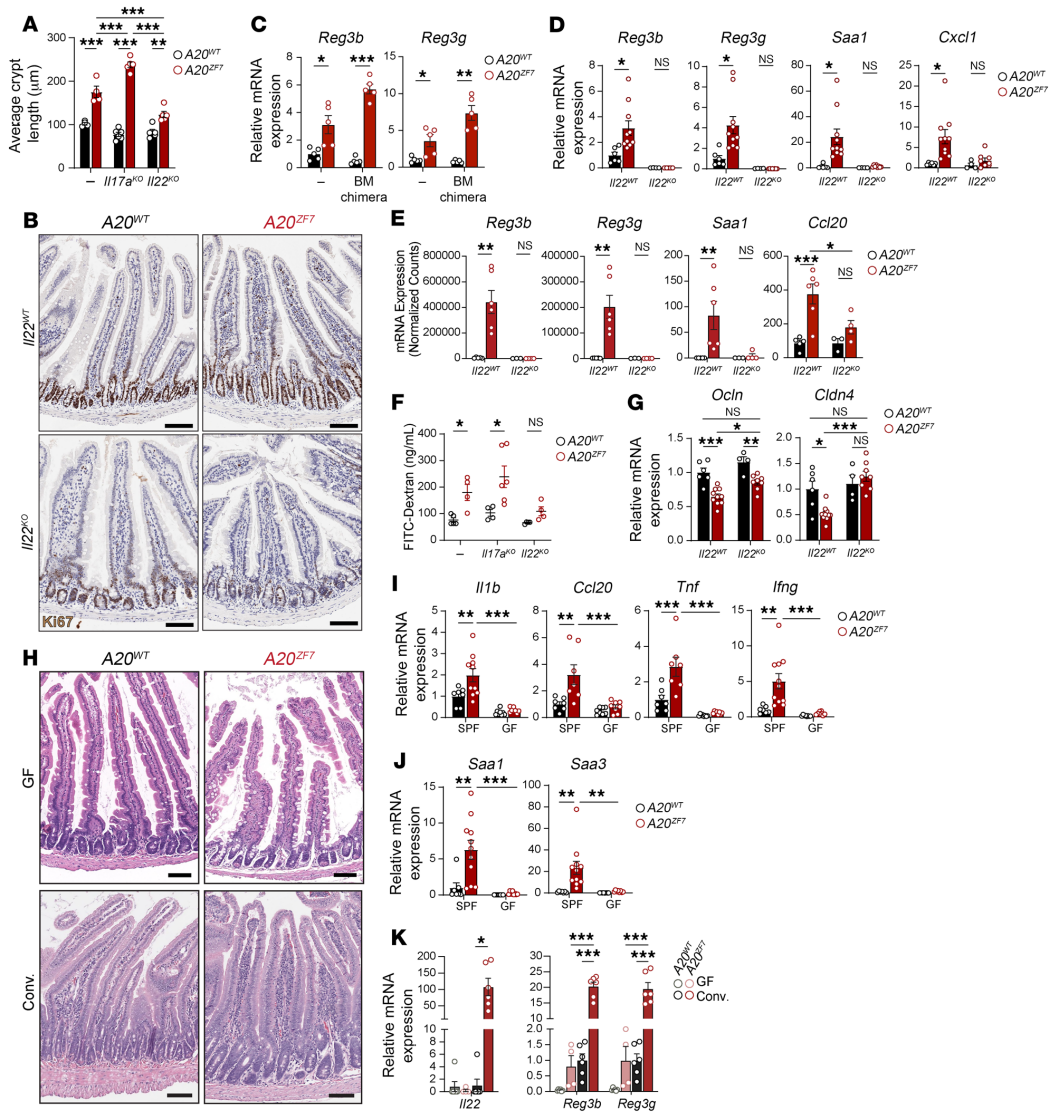
IL-22 disrupts epithelial barrier integrity and drives microbe-dependent enteritis in *A20^ZF7^* mice. (**A**) Average crypt length measured in histology sections of proximal small intestines from indicated genotypes of mice. (**B**) Representative immunohistochemistry of Ki67 expression in small intestines of indicated genotypes of mice. Data are representative of 2–3 mice from each genotype. Scale bars: 100 μm. (**C**) qPCR analyses of IL-22–dependent defensins from intact small intestines of indicated radiation chimeras. (**D**) qPCR analyses of IL-22–dependent genes from isolated small intestinal IECs. (**E**) Normalized mRNA counts of IL-22–dependent genes from bulk RNA-Seq of intact small intestines. (**F**) Fluorescence measurements of FITC-dextran in sera from indicated genotypes of mice 4 hours after FITC-dextran gavage. (**G**) qPCR analyses of indicated tight junction genes from isolated small intestinal IECs from indicated genotypes of mice. (**H**) Representative H&E histology of proximal small intestines from germ-free (GF) or conventionalized (Conv.) mice of indicated genotypes. Scale bars: 200 μm. (**I** and **J**) qPCR analyses of indicated genes from small intestines from indicated genotypes of specific pathogen–free (SPF) and GF mice. (**K**) qPCR analyses of indicated genes from small intestines from indicated genotypes of GF or Conv. mice. Data are shown as mean ± SEM. Each circle represents an independent mouse. Statistics were calculated using unpaired 2-tailed Student’s *t* test with Welch’s correction (**C**–**F**, *Il22* in **K**) or 2-way ANOVA with Bonferroni’s multiple-comparison correction (**A**, *Ccl20* in **E**, **G**, **I**, **J**, *Reg3b* and *Reg3g* in **K**). *P < 0.05, **P < 0.01, ***P < 0.001.

Il22ra1, the IL-22–specific receptor chain, is selectively expressed on intestinal epithelial cells (IECs) but not immune cells (28). Hence, pathophysiological effects of IL-22 in *A20^ZF7^* mice could be mediated by perturbation of IEC functions. By histology, epithelial crypts were disproportionally expanded in small intestines of *A20^ZF7^* mice when compared with WT mice (Figure 4A and Figure 5A). Bulk RNA-Seq highlighted epithelial cell proliferation as an enriched gene set in *A20^ZF7^* intestines (Figure 2A), and immunohistochemistry for Ki67 confirmed an expansion of proliferating crypt IECs in *A20^ZF7^* intestines (Figure 5, A and B). Interestingly, chimeras bearing *A20^ZF7^* hematopoietic cells also expressed elevated levels of IEC-derived defensins *Reg3b* and *Reg3g* (Figure 5C). This result supports that radiation-sensitive *A20^ZF7^* Th17 cells express exaggerated amounts of IL-22 that perturb WT IECs. Notably, the IEC proliferation and crypt elongation seen in *A20^ZF7^* mice were significantly lessened in *A20^ZF7^* *Il22^–/–^* mice (Figure 5, A and B). To understand the IL-22–dependent perturbations of IECs in *A20^ZF7^* mice, we isolated small intestinal IECs from *A20^ZF7^* *Il22^–/–^* and control mice. Transcriptomic analyses of these cells revealed that *A20^ZF7^* epithelia expressed more C-type lectins (*Reg3b*, *Reg3g*), alarmins (*Saa1*), and chemokines (*Cxcl1*) than WT IECs (Figure 5, D and E). These defects were markedly reduced in *A20^ZF7^* *Il22^–/–^* IECs, indicating that IL-22 drives exaggerated expression of these proinflammatory mediators in *A20^ZF7^* IECs.

As proinflammatory cytokines can perturb epithelial barrier functions (29–31), we tested intestinal epithelial permeability in *A20^ZF7^* *Il22^–/–^* mice and control mice by gavaging these mice with FITC-dextran. Increased absorption of FITC-dextran in sera of *A20^ZF7^* mice supports aberrant barrier integrity in these mice. This compromised barrier integrity was similarly exacerbated in *A20^ZF7^* *Il17a^–/–^* mice but normalized in *A20^ZF7^* *Il22^–/–^* mice (Figure 5F). To understand why *A20^ZF7^* IECs fail to maintain barrier integrity, we assayed expression of occludin (*Ocln*) and claudin-4 (*Cldn4*), 2 tight junction proteins that support epithelial barrier integrity (32, 33). *Ocln* and *Cldn4* expression were depressed in *A20^ZF7^* compared with WT IECs, and *Cldn4* was normalized in *A20^ZF7^* *Il22^–/–^* IECs (Figure 5G). Although *Ocln* in *A20^ZF7^* *Il22^–/–^* IECs did not reach levels similar to those in *Il22^–/–^* IECs, these were normalized to WT levels. Diminished barrier integrity may allow translocation of microbes and pathogenic products, inciting an inflammatory response (34–36). To determine whether intestinal microbiota are required for enteritis in *A20^ZF7^* mice, these mice were derived into germ-free environments. Germ-free *A20^ZF7^* mice exhibited neither intestinal inflammation (Figure 5H) nor elevated expression of proinflammatory cytokines (i.e., *Il1b*, *Ccl20*, *Tnf*, *Ifng*) relative to controls (Figure 5I), a stark contrast to the *A20^ZF7^* mice raised in specific pathogen–free (SPF) environments. Despite the mutation of *A20^ZF7^* in the small intestines of germ-free mice, the absence of commensal microbiota did not lead to alarmin (*Saa1*, *Saa3*) production (Figure 5J), suggesting that microbiota are necessary for IL-22–dependent programs within the small intestine. Segmented filamentous bacteria (SFB) preferentially colonize and induce Th17 responses in the terminal ileum but not duodenum (37–40). Specific PCR detection of SFB 16S rRNA in our mice infrequently showed SFB in ilea of WT and *A20^ZF7^* intestines (Supplemental Figure 3A) and never detected SFB in duodena from either genotype. Therefore, the duodenitis of *A20^ZF7^* mice is microbe dependent but unlikely to be driven by SFB.

As our SPF *A20^ZF7^* and WT mice were cohoused, thereby sharing luminal microbes via coprophagic behavior, *A20^ZF7^* intestines may have responded aberrantly to the same commensal microbes that were present in WT cagemates. To further test this notion, we harvested luminal commensal microbes from an unrelated colony of WT C57BL/6 mice, pooled these organisms, and introduced these microbes (“WT” microbes) into cohoused 6-week-old *A20^ZF7^* and WT germ-free mice. These neocolonized, or “conventionalized,” mice were maintained as cagemates for 6 weeks. Analyses of intestines from these mice revealed that duodenal tissues from conventionalized WT mice minimally expressed *Il22* and IL-22–dependent genes compared with those from germ-free WT mice (Figure 5K). By contrast, conventionalized *A20^ZF7^* mice expressed markedly elevated levels of these genes compared with germ-free *A20^ZF7^* mice or conventionalized WT mice (Figure 5K). Correspondingly, conventionalized *A20^ZF7^* mice exhibited marked duodenal, but not ileal or colonic, inflammation when compared with conventionalized WT mice (Figure 5H and Supplemental Figure 1D). Moreover, germ-free and conventionalized mice harbored no detectable SFB (Supplemental Figure 3B), further supporting that the duodenal inflammation in *A20^ZF7^* mice is microbe dependent but, also, independent of SFB. Taken together, these data indicate that *A20^ZF7^* intestines respond aberrantly to “WT” microbiota by expressing markedly elevated expression of IL-22 that, in turn, perturbs IEC functions, disrupts intestinal epithelial barrier integrity, and causes duodenitis.

### A20^ZF7^ restrains IL-22 expression in murine and human CD4^+^ T cells.

**Figure 6:**
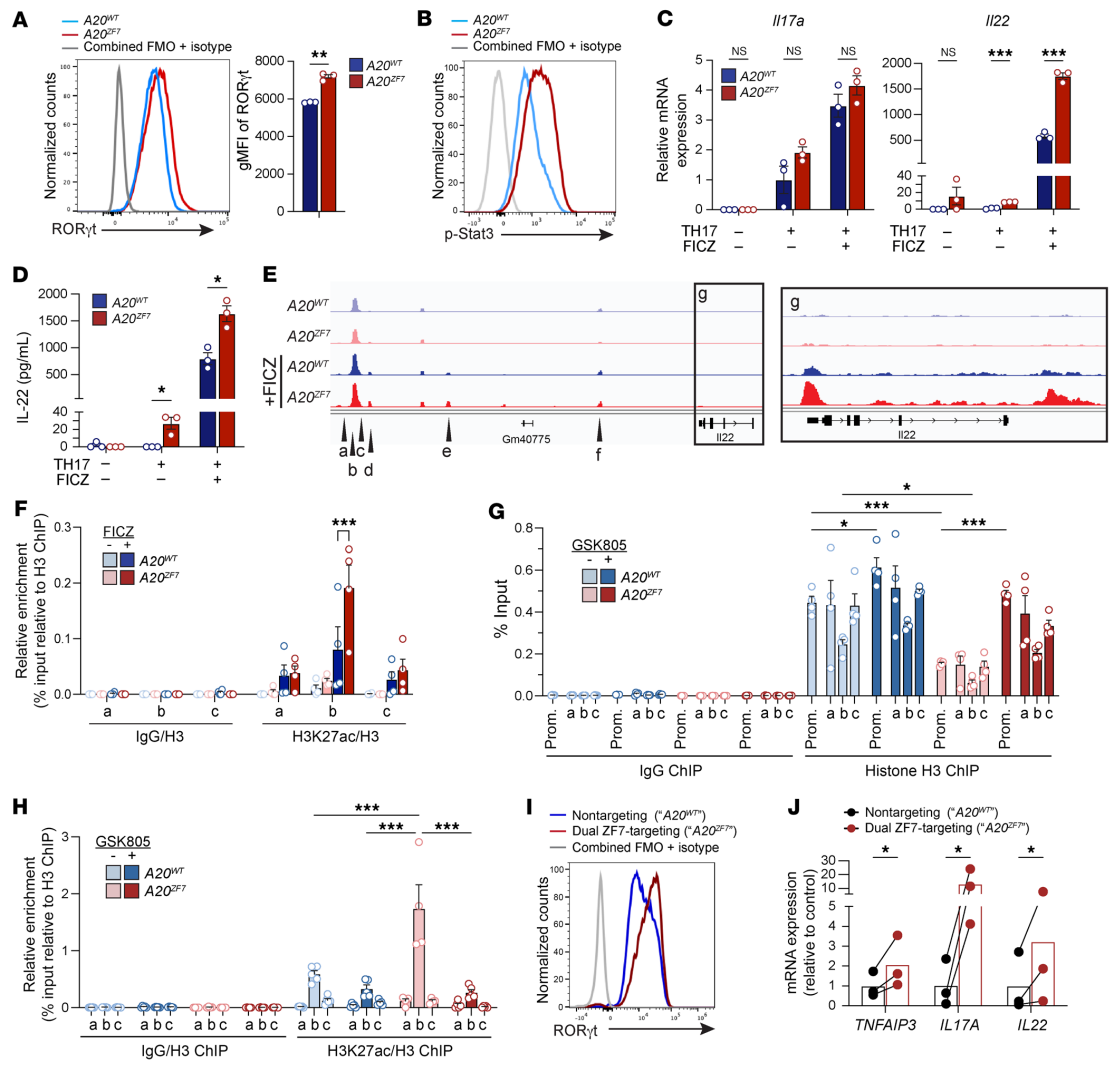
*A20^ZF7^* Th17 cells show enhanced IL-22 production due to increased histone acetylation at an *Il22* enhancer. (**A** and **B**) Flow nucleometry of RORγt (**A**) or phosphorylated Stat3 (Tyr705) (**B**) expression in nuclei isolated from in vitro–differentiated Th17 cells from WT and *A20^ZF7^* mice. Histograms are representative of *n* = 2–3 biologically independent experiments. (**C**) qPCR analyses of *Il17a* and *Il22* expression in cells generated as in **A**. “Th17” indicates Th17 differentiation conditions. “FICZ” indicates treatment with Ahr agonist FICZ. (**D**) ELISA of IL-22 secretion from cells generated as in **A**. (**E**) ATAC-seq of genomic loci at or near the *Il22* locus in cells generated as in **A**. (**F**) ChIP of acetylated H3K27 at indicated *Il22* loci (loci a–c in **E**) in cells generated as in **A**. (**G**) ChIP of histone H3 at the *Il22* promoter and enhancer loci (loci a–c in **E**). Cells were generated as in **A** and treated with the RORγt inhibitor GSK805. H3 ChIP directly assesses nucleosome occupancy and, thus, chromatin accessibility. (**H**) ChIP of acetylated H3K27 at *Il22* enhancer loci (loci a–c in **E**). Cells were generated as in **A** and treated with the RORγt inhibitor GSK805. (**I**) Flow cytometric analyses of RORγt expression in CRISPR/Cas9–edited primary human T cells differentiated in vitro using Th17 conditions. (**J**) qPCR analyses of expression of indicated genes in paired isogenic human Th17 cells engineered with CRISPR/Cas9 and either *A20^ZF7^*-targeted or nontargeting guide RNAs. Three pairs of isogenic samples from 2 healthy donors are shown. Data are shown as mean ± SEM. Statistics were calculated using unpaired 2-tailed Student’s *t* test with Welch’s correction (**C**, **D**, and **F**), 2-way ANOVA with post hoc Tukey’s multiple-comparison correction with simple effects (**G** and **H**), or paired-ratio *t* test (**J**). *P < 0.05, **P < 0.01, ***P < 0.001.

Our data above show that T cell–autonomous *A20^ZF7^* functions and IL-22 are integral to enteritis in *A20^ZF7^* mice. Accordingly, we investigated how *A20^ZF7^* regulates T cell production of IL-22. We enriched naive CD4^+^ T cells from 8-week-old *A20^ZF7^* and WT mice and differentiated these cells into Th17 cells with recombinant IL-6 and TGF-β. The efficiency of in vitro Th17 differentiation (as defined by the expression of RORγt, the key transcription factor that coordinates Th17 differentiation) (41) was consistently greater than 99% (Figure 6A), regardless of A20 mutation status. Notably, *A20^ZF7^* Th17 cells exhibited greater amounts of RORγt localized to the nucleus than WT Th17 cells, suggesting that *A20^ZF7^* restrains RORγt expression and/or nuclear localization in a cell-autonomous fashion (Figure 6A). *A20^ZF7^* Th17 cells also expressed more phosphorylated Stat3, reflecting substantial activation of this pathway (Figure 6B). In the absence of PMA/ionomycin stimulation, *A20^ZF7^* Th17 cells also expressed more *Il22* mRNA than WT cells (Figure 6C). Since *Il22* expression is dependent on the aryl hydrocarbon receptor (Ahr) (42), these cells were treated with the Ahr agonist FICZ (42, 43). FICZ further exaggerated enhanced *Il22* expression in *A20^ZF7^* Th17 cells relative to WT cells (Figure 6C). Similarly, ELISA of supernatants from these cells revealed that *A20^ZF7^* T cells secreted more IL-22 protein than WT cells (Figure 6D). Notably, these results were obtained from cells that were not stimulated with PMA or ionomycin, avoiding potential caveats associated with possible A20-dependent regulation of these stimuli. Aligned with these findings, flow cytometry studies of PMA/ionomycin-stimulated cells showed increased numbers of IL-22^+^ T cells in *A20^ZF7^* cultures when compared with WT cultures (Supplemental Figure 4B). Hence, mutation of *A20^ZF7^* within T cells leads to greater RORγt expression and increased expression of IL-22.

As *A20^ZF7^* Th17 cells express more *Il22* mRNA and protein than WT cells, we hypothesized that *A20^ZF7^* restrains epigenetic regulation of *Il22* transcription. To identify potential genomic loci that may regulate *Il22* in *cis*, we surveyed chromatin accessibility via assay for transposase-accessible chromatin sequencing (ATAC-seq) followed by massively-parallel sequencing of Th17 cells differentiated from naive WT and *A20^ZF7^* CD4^+^ T cells. ATAC-seq revealed increased accessibility across the *Il22* gene in *A20^ZF7^* Th17 cells (Figure 6E, region g), supporting open chromatin at *Il22*’s promoter and enhanced *Il22* transcription. In addition, multiple other sites upstream of *Il22* demonstrated DNA accessibility, predicting potential enhancer regions (Figure 6E, regions a–f). Notably, ATAC-seq highlighted a conserved region 32 kb upstream of *Il22*’s transcription start site (Figure 6E, region b), a site recently characterized as an *Il22* enhancer (44). To define this conserved region as a bona fide functional enhancer and to quantify the activation state of this enhancer, naive WT and *A20^ZF7^* CD4^+^ T cells were differentiated into Th17 cells, and chromatin immunoprecipitation (ChIP) of acetylated lysine 27 of histone H3 (H3K27ac) was performed at these genomic loci. H3K27ac was increased in *A20^ZF7^* Th17 cells at the enhancer for *Il22* (Figure 6F, region b), supporting increased enhancer activation and enhanced *Il22* transcription in *A20^ZF7^* Th17 cells. By contrast, sequences located on the immediate shoulders of the enhancer (Figure 6F, regions a and c) as well as other DNA accessible sites (Supplemental Figure 4C, regions d–f) were less decorated with H3K27ac and similarly marked in WT and *A20^ZF7^* Th17 cells. The enhancement of H3K27ac marks at the conserved enhancer with FICZ treatment suggests that Ahr activation facilitates the acetylation of this locus. These results provide a molecular underpinning for Ahr in promoting *Il22* expression (42, 43). As our flow studies of isolated nuclei showed that *A20^ZF7^* Th17 cells harbor increased accumulation of nuclear RORγt compared with WT Th17 cells (Figure 6A), we next tested whether this increased RORγt mediates increased transcriptional activity at the *Il22* enhancer. We differentiated naive CD4^+^ WT and *A20^ZF7^* cells into Th17 cells in vitro and acutely treated cells with GSK805 (a RORγt-specific inhibitor) (45, 46) overnight beginning on day 6 of differentiation. Subsequent chromatin analyses at the enhancer showed that RORγt inhibition in *A20^ZF7^* Th17 cells normalized nucleosome occupancy/density (Figure 6G) and H3K27ac (Figure 6H) to WT levels. These findings support RORγt’s role in reducing nucleosomal occupancy, facilitating chromatin and DNA accessibility, and enhancing acetylation of H3K27 at the *Il22* enhancer in *A20^ZF7^* Th17 cells. Therefore, elevated RORγt in differentiated *A20^ZF7^* Th17 cells actively remodels chromatin at the *Il22* enhancer to drive increased *Il22* transcription. Taken together, these studies suggest that *A20^ZF7^* restrains RORγt activity to limit chromatin accessibility and hyperacetylation of a specific *Il22* enhancer, thus restricting *Il22* transcription in CD4^+^ Th17 cells.

The *TNFAIP3* gene is well conserved between murine and human genomes, and A20 polymorphisms and mutations are associated with human IBD and celiac disease (7, 10, 47). To determine whether A20’s ZF7 domain regulates Th17 cell functions and IL-22 expression in human T cells, we generated *A20^ZF7^* mutant human CD4^+^ T cells using CRISPR/Cas9. Since A20’s ZF7 domain comprises the most C-terminal residues of the A20 protein, targeting *A20^ZF7^* is unlikely to affect the stability or structure of the A20 protein. Our recent studies of murine *A20^ZF7^* mutant proteins suggest that these proteins are indeed expressed at supranormal levels in TNF-stimulated fibroblasts (21). We thus designed CRISPR guide RNAs (gRNAs) to delete the N-terminal half of *A20’s ZF7* domain. We isolated naive CD4^+^ T cells from healthy donor peripheral blood, activated the cells via TCR stimulation, and electroporated CRISPR/Cas9 along with either these ZF7-targeting or control gRNAs to generate *A20^ZF7^* mutant and isogenic control human T cells. Sanger sequencing demonstrated that more than 85% of alleles were targeted by *A20^ZF7^*-specific gRNAs (Supplemental Figure 5, A and B). These cells were then differentiated toward Th17 cells for 9 days, at which time more than 99% expressed RORγt in both *A20^ZF7^*-mutated and control cells (Figure 6I), confirming efficient Th17 differentiation. Expression of *Tnfaip3* mRNA is markedly induced by NF-κB activity, and A20 mediates negative feedback on NF-κB signaling (26). In keeping with the compromised ability of *A20^ZF7^* cells to restrain NF-κB signaling, expression of *TNFAIP3* mRNA was increased in *A20^ZF7^* human T cells when compared with paired isogenic control T cells (Figure 6J). The NF-κB family members c-Rel and Rela/p65 bind RORγt promoters and drive RORγt transcription (48). Consistent with increased NF-κB signaling and with our findings in murine Th17 cells, ablation of *A20^ZF7^* in human Th17 cells led to higher expression of RORγt (Figure 6I). Finally, *A20^ZF7^*-deleted human Th17 cells led to elevated expression of *IL17A* and *IL22* transcripts relative to paired, isogenic, A20-competent control cells (Figure 6J). Hence, A20’s ZF7 motif restrains RORγt expression and *IL22* expression in human Th17 cells.

## Discussion

Our studies uncover a new spontaneous model of proximal enteritis that links A20’s M1-Ub–binding motif to pathogenic Th17 cell activation, IL-22–dependent epithelial dysfunction, microbe-dependent enteritis, and epigenetic regulation of *Il22* transcription. Inflammation of the proximal duodenum in *A20^ZF7^* mice aligns with two human diseases that can afflict this portion of the intestine: Crohn’s disease and celiac disease. Both of these diseases are genetically linked to extragenic polymorphisms upstream of *TNFAIP3* (7, 9). While the mechanisms by which A20 regulates intestinal immunity remain incompletely understood, we show here that non-enzymatic Ub binding by A20 ZF7 is more important than A20 ZF4’s Ub binding or E3 Ub ligase activity for preserving small intestinal homeostasis. The selective importance of A20 ZF7’s Ub-binding motif may be related to its preferential binding to M1-linked Ub dimers (23), its increased binding affinity to Ub tetramers (22), and/or its ability to bind and regulate IKKγ signaling complexes (21). While the exact *Tnfaip3* mutation we have engineered in A20’s ZF7 motif has not been described in human patients, haploinsufficient HA20 patients harbor many *TNFAIP3* mutations that cause premature stop codons that commonly result in the loss of A20’s C-terminal ZF7 domain (10, 47, 49). Therefore, our findings not only highlight the importance of the non-enzymatic ZF7 functions of the A20 protein in intestinal immune homeostasis but also identify a unifying molecular dysfunction that may regulate intestinal disease in human patients.

We have obtained new insights into the homeostasis of intestinal tissue-resident Th17 cells. Expansion of Th17 cells in *A20^ZF7^* mouse intestines may reflect exaggerated TCR responses and increased IL-1β expression. Aberrant TCR and cytokine responses likely drive Th17 cells toward proinflammatory states characterized by the expression of IL-17F, IL-22, GM-CSF, M-CSF, and/or granzymes (50, 51). This important transition can be stimulated by IL-23 (50, 52). The importance of Th17-derived cytokines other than IL-17A in the intestine has been implicated by the clinical antiinflammatory efficacy of IL-23 blockade contrasted with proinflammatory consequences of IL-17A inhibition (53). Our studies highlight the ability of IL-22 to drive proximal enteritis. Increased expression of other Th17 cytokines in *A20^ZF7^* mouse intestines, e.g., IL-17F and/or IFN-γ, may also contribute to enteritis in these mice. More broadly, increased cytokine expression by intestinal *A20^ZF7^* T cells implicates *A20^ZF7^* as a critical mediator of Th17 cell quiescence.

In addition to supporting epithelial cell proliferation and repair, our studies show that IL-22 can also disrupt IEC expression of tight junction proteins and alarmins. Among cytokines that directly regulate IEC functions, IL-22 is distinct from IL-17A, IL-17F, and IFN-γ in that IL-22 binds to IECs and not immune cells (28). In this regard, IL-22 occupies a unique niche in immune-epithelial crosstalk. While prior studies showed that IL-22 supports reparative functions in IECs (2, 54, 55), IL-22 can also mediate inflammation when Treg or IL-10 deficiency causes aberrant macrophage activation and colitis (56). By contrast, *A20^ZF7^* mice develop enteritis in the presence of supranormal levels of Tregs and IL-10. Hence, our studies reveal that IL-22 can cause intestinal inflammation despite intact Treg functions. IL-22–dependent pathophysiology in *A20^ZF7^* mice also appears distinct from that seen in Treg- and IL-10–deficient mice in that *A20^ZF7^* mice develop proximal enteritis, while the latter models predominantly develop colitis. We have found that IL-22 stimulates exaggerated IEC elaboration of alarmins, hyperproliferation of crypt IECs, and compromised epithelial permeability, leading to microbe-dependent enteritis. These epithelial dysfunctions broaden IL-22–dependent biology beyond its tissue-reparative and defensin activities. They demonstrate that dysregulated IL-22 expression from pathogenically activated Th17 cells can profoundly disrupt IEC homeostasis. The context-specific variables that influence whether IL-22 performs proinflammatory versus tissue-reparative functions may include the cellular source of IL-22 (i.e., Th17 versus ILC3 cells), the epithelial subtypes responding to IL-22 (e.g., small versus large intestine, crypt progenitor versus mature epithelial cells), coexpressed cytokines, and/or the abundance or duration of IL-22 signals.

We have found that enteritis in *A20^ZF7^* mice is microbe dependent. Yet this enteritis selectively afflicts the proximal small intestine that is typically colonized with fewer numbers of bacteria than more distal regions. Our studies with neocolonized, or “conventionalized,” *A20^ZF7^* mice most directly establish the microbe-dependence of our enteritis model and provide a platform for future dissection of microbe-driven responses in these mice. Segmented filamentous bacteria (SFB) selectively expand Th17 cells in ilea of mice, and this selectivity is related to this organism’s preferential colonization of this segment of the intestine (37–40). Indeed, we detected SFB in the ilea — but not the duodena — of a few WT and *A20^ZF7^* mice. Hence, it is more likely that microbes that selectively colonize the proximal small intestine may drive duodenitis in *A20^ZF7^* mice. Small-intestinal microbes have recently been highlighted to alter lipid absorption (57). Alternatively, IECs in the proximal small intestine of these mice may harbor preferential sensitivity to microbially triggered IL-22 signals. Other potential contributing factors include luminal products that are concentrated in the proximal bowel, e.g., bile acids emptied into the duodenum via the bile duct. As we have found that germ-free conditions abrogate enteritis, these moieties could be secondary bile acids that are modified by small intestinal bacteria. Future studies of duodenal microbes and/or bile acid metabolites could unveil mechanisms by which homeostasis is preserved in the duodenum.

We have uncovered T cell–autonomous functions of A20 ZF7 that restrain pathogenic expression of IL-22. We have observed increased STAT3 phosphorylation in in vitro–differentiated *A20^ZF7^* Th17 cells, suggesting that *A20^ZF7^* may restrain IL-6/IL-21–induced JAK/STAT signals in these cells. Prior work from our laboratory and others showed that A20 restrains TCR-induced NF-κB signaling in naive T cells (15, 18, 58). Hence, intestinal *A20^ZF7^* Th17 cells may exhibit enhanced NF-κB signaling in response to homeostatic TCR signals. Notably, the enhanced *Il22* transcription in in vitro–differentiated *A20^ZF7^* Th17 cells occurs in the absence of IL-1 or IL-23, implicating *A20^ZF7^* in the restraint of Th17 cell responses independent of these pathogenic cytokines. Prior studies showed that components of NF-κB — c-Rel and Rela/p65 — directly promote the expression of RORγt (48). Our current results are consistent with the idea that increased NF-κB activity in *A20^ZF7^* Th17 cells stimulates increased RORγt expression. Our data further indicate that increased nuclear RORγt directly promotes *Il22* enhancer activity that enhances *Il22* transcription in *A20^ZF7^* T cells. This enhancer has been described to harbor NF-κB, RORγt, Runx1, and AP-1 binding sites (44). Intriguingly, this murine enhancer is also highly conserved upstream of the human *IL22* locus. Combined with our finding that ablation of *A20^ZF7^* in human T cells causes increased *IL22* expression, inhibition of this enhancer by *A20^ZF7^* prevents proinflammatory expression of *IL22* and may restrain a program of pathogenic activation of both murine and human Th17 cells Given the importance of pathogenically activated Th17 cells to intestinal inflammation, this biochemical function provides an important new lever for preserving Th17 cell quiescence and preventing human disease.

## Methods

### Sex as a biological variable.

Both male and female mice were used in this study, and similar results were obtained with both. Sex was not considered a biological variable in this study.

### Mice.

Animal studies were conducted under an approved Institutional Animal Care and Use Committee protocol at UCSF. Mice were bred and housed at 22°C under a 12-hour light/12-hour dark cycle with ad libitum access to food and water. *A20^C103/C103^* (OTU), *A20^ZF4/ZF4^* (ZF4), and *A20^ZF7/ZF7^* (ZF7) knockin mice were generated in our laboratory and previously described (20, 21). B6.129S2 Ighm^tm1Cgn^/J (μMT; 002288), B6.129S7 RAG-1^tm1Mom^/J (Rag1; 002216), and C57BL/6 Il22^tm1.1(icre)Stck^/J (IL22; 027524) mice were purchased from The Jackson Laboratory. *Il17a^–/–^* mice were provided by Y. Iwakura (Tokyo University of Science, Tokyo, Japan) via S. Gaffen, University of Pittsburgh, Pittsburgh, Pennsylvania, USA).

### Tissue histology and scoring.

Mice at the indicated ages were euthanized, and the proximal small intestine was collected in 4% paraformaldehyde. Samples were subsequently processed and stained by HistoWiz to produce H&E-, CD4-, or Ki67-stained slides. Pathology scores of intestinal inflammation were generated by assessment of total tissue inflammation and epithelial changes in the colon. The total inflammation score (scale 0–3) was based on the overall severity or extent of inflammation in the intestine. An epithelial change score (scale 0–4) was assigned for each of the following categories: goblet cell loss, intraepithelial neutrophils and/or cryptitis, abscesses, and crypt loss. The scores were summed to generate a pathology score for each mouse.

### Radiation bone marrow chimeras.

At 6 weeks of age, WT recipient mice were irradiated with 12 Gy total-body radiation. The same day, recipients were injected intravenously with 5 × 10^6^ bone marrow cells isolated from femora and tibiae of corresponding donors (WT or *A20^ZF7^* mice). Animals were kept on antibiotics in the drinking water for 1 week after irradiation. Bone marrow recipients were sacrificed 12 weeks after bone marrow transfer.

### FITC-dextran epithelial barrier assay.

Mice (11 weeks old) were fasted for 5 hours before oral gavage of FITC-dextran (average MW 4,000; Sigma-Aldrich 46944) at 500 mg/kg body weight. Serum was collected 4 hours after gavage, and the fluorescence intensity was measured on a Molecular Devices fluorescence microplate reader.

### Isolation of lamina propria cells.

Single lamina propria cells were isolated from small intestinal lamina propria as previously described (59). Briefly, the proximal 10 cm of small intestines from euthanized mice were flushed with cold PBS to clear feces and mucus. Excess mesenteric fat and Peyer patches were removed. Intestines were opened longitudinally and incubated in pre-digestion buffer (calcium- and magnesium-free HBSS, 3% FBS, 10 mM HEPES [pH 7.4], 1 mM dithiothreitol, 5 mM EDTA) twice for 15 minutes each, rinse buffer (calcium- and magnesium-free HBSS, 3% FBS, 10 mM HEPES [pH 7.4]) once for 5 minutes, and digestion buffer (RPMI with 3% FBS, 10 mM HEPES [pH 7.4], 0.03 mg/mL DNase I, 0.1 mg/mL Liberase TM (Sigma 5401119001) for 10 minutes, all at 37°C with agitation at 220 rpm. Enzyme-digested tissue was further dissociated in a gentleMACS C-tube using the gentleMACS (Miltenyi Biotec) Dissociator m_intestine program, added to RPMI with 10 mM HEPES (pH 7.4) and 3% FBS, and filtered through 70- or 100-μm filters. Leukocytes were enriched from the interface of a 40%/80% Percoll (GE Healthcare 17-081-01) gradient after centrifugation.

### Flow cytometry.

Single-cell suspensions were twice rinsed with PBS, stained with an amine-reactive viability dye in PBS for 15 minutes, quenched, and washed with FACS wash buffer (FWB: HBSS or PBS with 0.5% BSA). Cells were then blocked with 2 μg FcBlock (per 1 million cells) for 15 minutes and stained with antibodies against cell surface antigens for 30 minutes. For nuclear assays, nuclei were isolated as previously described (60) with slight modifications: cells were incubated in ice-cold isolation buffer (375 mM sucrose, 10 mM HEPES [pH 7.9], 10 mM potassium chloride, 5 mM magnesium chloride, 0.1% vol/vol Triton X-100, protease and phosphatase inhibitors) on ice for 15 minutes, and nuclei were collected by centrifugation at 1,300*g* for 5 minutes at 4°C. For intracellular antigens, cells/nuclei were subsequently fixed in 5% neutral-buffered formalin for 30 minutes at room temperature, permeabilized with eBioscience Perm Buffer (Thermo Fisher Scientific 00-8333-56), blocked with normal rat serum (STEMCELL Technologies) for 15 minutes, and rocked with primary antibodies overnight at 4°C. Single cells/nuclei were washed twice with FWB before analysis on a flow cytometer.

The following antibodies were used for flow cytometry: anti-CD45 (30-F11, BD Biosciences), anti-CD90.2 (30-H12, BD Biosciences), anti-TCRβ (H57-597, BioLegend), and anti-CD4 (GK1.5, BioLegend) for staining of mouse cell surface proteins; and anti–IL-17A (TC11-8H4, BioLegend), anti–IL-22 (poly1564, BioLegend), anti-RORγt (B2D, Thermo Scientific; or REA278, Miltenyi Biotec), and anti–phosphorylated Stat3 (4/P-STAT3, BD Biosciences) for staining of human or mouse intracellular proteins. For intracellular cytokine staining, cells were first incubated for 4 hours in medium with 100 ng/mL phorbol 12-myristate 13-acetate (PMA), 1 μg/mL ionomycin, and 5 μg/mL brefeldin A.

### Murine in vitro Th17 differentiation.

Naive CD4^+^ T cells were isolated from murine splenocytes using a Mouse CD4 naive T cell negative selection kit (STEMCELL Technologies 19765) per the manufacturer’s instructions. Isolated cells were subsequently differentiated in plates precoated with 2 μg/mL anti-CD3 (overnight at 4°C, or 2 hours at 37°C) in Th17 medium (IMDM with 10% FBS, 50 μM β-mercaptoethanol, 2 μg/mL anti-CD28 [Bio X Cell BE0015, clone 37.51], 20 ng/mL recombinant murine IL-6 [R&D Systems 406-ML], 5 ng/mL recombinant human TGF-β1 [PeproTech 100-21], 10 μg/mL anti–IL-4 [Bio X Cell BE0045, clone 11B11], and 10 μg/mL anti–IFN-γ [Bio X Cell BE0055, clone XMG1.2]) at a density of 1 million cells/mL. Medium was replenished on day 2, and cells were split into fresh medium on day 3 of differentiation to maintain a cell density of 1–2 million cells/mL. For RORγt inhibition experiments, naive CD4^+^ T cells were differentiated for 6 days before incubation with 1 μM GSK805 (MedChemExpress HY-12776) in fresh Th17 medium for 16 hours prior to harvesting.

### RNA isolation and quantitative real-time PCR.

Total RNA was isolated from whole intestinal tissue or single-cell suspensions (epithelial fractions or lamina propria) using QIAGEN RNeasy extraction kits or TRIzol (Invitrogen) per the respective manufacturer’s instructions. Whole tissues were lysed in either ice-cold TRIzol or QIAGEN RLT Buffer using Lysing Matrix D (MP Biomedical 116913050-CF) with MP Biomedical FastPrep-24 Homogenizer (1 cycle of 20 seconds at 4 m/s). Single-cell suspensions were lysed directly in TRIzol. RNA was reverse-transcribed using High Capacity cDNA Reverse Transcription kit (Thermo Fisher Scientific 4368814) per the manufacturer’s instructions and assayed by quantitative real-time PCR using TaqMan probes (Thermo Scientific) or the SYBR Green (Thermo Scientific) method. Relative mRNA expression was calculated using the Livak (ΔΔCt) method, with *Actb* as a loading control, unless otherwise noted. In experiments using human cells, the housekeeping genes *ACTB*, *GAPDH*, *EIF4G2*, and *RPLP0* were collectively used, and the geometric mean was used in calculating relative mRNA expression. To detect segmented filamentous bacteria (SFB), 16S ribosomal RNA (rRNA) was amplified using primer sequences specific to SFB 16S rRNA: 5′-GATCCTGGCTCAGGACGAAC-3′ and 5′-TTCATCGGGCTATCCCCCA-3′. PCR products from ileal samples with detectable levels of SFB 16S rRNA were subjected to agarose electrophoresis, and the 146-bp products were excised, purified using QIAGEN Gel Extraction kit, and Sanger-sequenced. NCBI BLAST of Sanger sequences confirmed the PCR to specifically detect SFB 16S rRNA. 16S rRNA copies were calculated based on a standard curve generated using a double-stranded DNA gBlock template (Integrated DNA Technologies) encoding a portion of the SFB 16S rRNA sequence. The limit of detection of this assay is routinely less than 1 copy of SFB 16S rRNA.

### Single-cell RNA sequencing and data analysis.

Lamina propria cells were isolated from small intestines of 2 mice of each genotype as described above. Isolated cells were negatively selected for T cells and ILCs (STEMCELL Technologies 19851) and stained with TotalSeq-C anti-mouse hashtagged antibodies (BioLegend). Cells from each animal were separately hashtagged. For each genotype, a total of 60,000 cells were collected for processing using the 10x Genomics Chromium Next GEM Single Cell 5′ Kit v1.1. Sequences were aligned and processed with Cell Ranger v7.1 using the mm10 reference genome and default parameters. Cell Ranger output was further processed with R version 4.3.3 and Seurat version 4.3 (49). Seurat objects were created using only genes that appeared in at least 3 cells. Cells were further filtered to exclude multiplets (defined as having 2 or more different hashtags) and low-quality/multiplet cells (cells with fewer than 200 detected genes, more than 2,500 detected genes, or more than 15% mitochondrial reads). Read counts were then normalized using NormalizeData. After viewing of UMAP clusters, T and innate lymphocyte clusters were selected based on the presence of T/innate lymphocyte genes (*Ptprc*, *Trac*, *Trdc*, *Cd3e*, *Cd4*, *Cd8a*, *Ncr1*, *Klrb1b*) as well as the absence of non-T/non-innate lymphocyte genes (*Des*, *Acta2*, *Col1a2*, *Pecam1*, *Cdh5*, *Epcam*, *Cd79a*, *Mcpt1*, *Apoe*).

Cell clusters were generated using the Louvain algorithm implemented by the FindClusters function. Marker genes for each cluster were determined using Wilcoxon’s test on the raw counts, implemented by the function FindAllMarkers, and clusters of cell types were additionally determined by manual inspection of the lists of cluster marker genes. Dimensionality reduction by UMAP was performed using the RunUMAP function with the 30 largest principal components. Visualization of all scRNA-Seq data was generated using the Seurat package and/or ggplot2. To remove cell cycle phases as a source of clustering heterogeneity, Seurat’s CellCycleScoring function was used to score each cell and regress out the effects of cell cycle genes/phases.

### Bulk RNA-Seq library preparation and analyses.

Total RNA was isolated from intact proximal small intestine as described above. One microgram of total RNA was depleted of rRNA (NEBNext rRNA Depletion Kit v2, New England Biolabs E7405) and subsequently fragmented, reverse-transcribed into cDNA, and amplified into barcoded libraries with NEBNext RNA Library Prep (New England Biolabs E7765) using custom barcoded Illumina-compatible primers. Libraries were pooled and sequenced on an Illumina NovaSeq X Plus instrument as 150-bp paired-end reads.

Sequenced reads (40 million to 70 million paired reads per sample) were processed with fastp (61) for quality control and adapter sequence trimming. Trimmed reads were aligned to the *Mus musculus* mm10 genome using HISAT2 (62), and transcriptomes for each sample were assembled with StringTie2 (63) and UCSC’s mm10 genes.gtf annotation. After construction of non-redundant transcriptomes across all samples (stringtie --merge), expression counts were tabulated (stringtie -e) and used as input for downstream gene expression analyses. DESeq2 (64) was used for differential gene expression analyses.

### Chromatin immunoprecipitation.

In vitro–differentiated Th17 cells were processed for chromatin immunoprecipitation (ChIP) as previously described (65). Briefly, 10 million Th17 cells were cross-linked at room temperature in 1% formaldehyde for 8 minutes, quenched with 125 mM glycine for 5 minutes, and resuspended in ChIP lysis buffer (10 mM Tris [pH 8], 1 mM EDTA, 0.5 mM EGTA, 0.5% wt/vol *N*-lauroyl sarcosine, and protease and phosphatase inhibitors). Each 250- to 300-μL aliquot was individually sonicated in a Bioruptor Pico to generate an average DNA fragment length of 250 bp as determined by agarose electrophoresis. Debris was spun down at 20,000*g* for 15 minutes, and the cleared chromatin supernatants were quantified and used for subsequent ChIP experiments. Sonicated chromatin was diluted to 500 μL in ChIP lysis buffer with 1% Triton X-100, 0.1% sodium deoxycholate, 1 mM EDTA, and protease and phosphatase inhibitors. Chromatin was precleared with 10 μL protein G Dynabeads (Thermo Fisher Scientific 10003D) for 4 hours at 4°C before incubation with 1 μg of primary antibodies overnight at 4°C. For anti–histone H3 (Abcam ab1791) or anti–acetyl histone H3 lysine 27 (anti-H3K27ac; Active Motif 39133) ChIP, 3 or 10 μg of total chromatin, respectively, was used. Protein G Dynabeads were incubated with immune complexes for 2 hours at 4°C before magnetic capture. Immunoprecipitates were washed 5 times with 1 mL RIPA wash buffer (50 mM HEPES [pH 7.6], 10 mM EDTA, 0.7% wt/vol sodium deoxycholate, 1% vol/vol IGEPAL CA-630 (Sigma-Aldrich), 0.5 M lithium chloride, and protease inhibitors) and once with TE (10 mM Tris with 1 mM EDTA [pH 8]) with 50 mM sodium chloride and eluted in elution buffer (10 mM Tris, 1 mM EDTA, 1% wt/vol sodium dodecyl sulfate, pH 8) at 56°C for 15 minutes. To reverse cross-links, eluates were incubated overnight at 65°C. Samples were diluted 2-fold in TE and sequentially digested with 80 μg of DNase-free RNase A at 37°C for 1 hour, and then 80 μg proteinase K at 56°C for 1 hour. ChIP DNA was purified by phenol/chloroform/isoamyl alcohol (25:24:1) and ethanol precipitation. DNA pellets were resuspended in 10 mM Tris (pH 8) and assayed by quantitative real-time PCR using the SYBR Green method. Antibodies were titrated to ensure they were not limiting.

### ATAC-seq library preparation and data analysis.

In vitro–differentiated Th17 cells were used to generate Tn5-tagmented DNA fragments as previously described (66). Briefly, to isolate nuclei, cells were lysed in ATAC lysis buffer (10 mM Tris-HCl [pH 7.5], 10 mM sodium chloride, 3 mM magnesium chloride, 0.1% vol/vol IGEPAL CA-630, 0.1% vol/vol Tween-20, 0.01% wt/vol digitonin) for 3 minutes on ice. The nuclei were briefly rinsed in wash buffer (10 mM Tris-HCl [pH 7.5], 10 mM sodium chloride, 3 mM magnesium chloride, 0.1% vol/vol Tween-20) before incubation in transposition buffer (10 mM Tris-HCl [pH 7.6], 5 mM magnesium chloride, 10% vol/vol dimethyl formamide, 0.1% vol/vol Tween-20, 0.01% wt/vol digitonin) with 100 nM loaded hyperactive Tn5 transposase (Diagenode C01070012) for 30 minutes at 37°C at 1,000 rpm. Transposed DNA was isolated from QIAGEN MinElute columns. Libraries were generated using published Illumina-compatible indexed oligonucleotides and 8 cycles of PCR amplification using NEBNext High-Fidelity 2X PCR Master Mix (New England Biolabs M0541). Libraries were purified from SPRIselect beads (Beckman Coulter B23317), and 51-bp paired-end sequences were sequenced on a NovaSeq X instrument. Sequences were aligned to the GRCm38/mm10 mouse reference genome using Bowtie 2 v2.4.5 (67), and PCR duplicates were removed by SAMtools v1.15 (68) followed by Picard v2.27.1 MarkDuplicates (https://broadinstitute.github.io/picard/). BigWig files were generated using UCSC’s bedGraphToBigWig script. DNA accessible regions and sites of differential accessibility were determined by MACS2 v2.2.7.1 (69) (https://pypi.org/project/MACS2/).

### CRISPR/Cas9 editing of human CD4^+^ T cells and in vitro Th17 differentiation.

Naive CD4^+^ T cells were isolated from healthy donor PBMCs using a human CD4 Naive T Cell negative selection kit (BioLegend 480041) per the manufacturer’s instructions. Isolated cells were stimulated in stimulation medium (Immunocult XF serum-free medium plus 50 μM β-mercaptoethanol, Immunocult anti-CD3/-CD28 cocktail [STEMCELL Technologies], and 50 IU/mL human recombinant IL-2 [NIH Biological Resources Branch. Preclinical Biologics Repository]) for 2–3 days. Stimulated cells were subsequently electroporated in a total of 20 μL P3 solution (Lonza) with Cas9 ribonucleoprotein complexes (3.1 μM TruCut Cas9 v2 [Thermo Fisher Scientific] with 9 μM CRISPR guides [Integrated DNA Technologies]) using the Lonza Amaxa 4D-Nucleofector and program EH-115. Target sequences recognized by CRISPR guides were GATACGTCGGTACCGGACCG for the control/non-targeting guide and TTTGGCAATGCCAAGTGCAA and ACCCCCCCAAGCAGCGTTGC for *A20^ZF7^* ablation. Electroporated cells were immediately placed into pre-warmed stimulation medium and incubated for 3 days. Genomic editing was confirmed to be at least 80% efficient by Sanger sequencing. Cells were subsequently differentiated into Th17 cells in plates precoated with 5 μg/mL anti-CD3 (Bio X Cell BE0001-2, clone OKT3) in Th17 medium (Immunocult XF serum-free medium with 50 μM β-mercaptoethanol, 30 ng/mL recombinant human IL-6 [Proteintech HZ-1019], 2.5 ng/mL recombinant human TGF-β1 [PeproTech 100-21], 10 ng/mL recombinant human IL-1β [Proteintech HZ-1164], 10 ng/mL recombinant human IL-23 [Proteintech HZ-1254], 10 μg/mL anti–IL-4 [Bio X Cell BE0240, clone MP4-25D2], and 10 μg/mL anti–IFN-γ [Bio X Cell BE0235, clone B133.5]) at a density of 1 million cells/mL. Cells were split and medium replenished on days 3, 6, and 8 of Th17 differentiation to maintain a cell density of 1–2 million cells/mL. Cells were harvested on day 9 of differentiation for analysis.

### Statistics.

Statistical analyses were performed using Prism 10 (GraphPad Software). Pathology scores were assessed by an unpaired Mann-Whitney *U* test. Quantitative real-time PCRs were assessed by a 2-tailed unpaired Student’s *t* test with Welch’s correction, unless otherwise noted. A significance level of 0.05 was used as the threshold for statistical significance. All data shown are representative of at least 2 independent experiments.

### Study approval.

All animal studies were conducted under an approved Institutional Animal Care and Use Committee protocol at the University of California, San Francisco (UCSF).

### Data availability.

Raw bulk RNA-Seq, scRNA-Seq, and ATAC-seq datasets generated for this study were deposited under Gene Expression Omnibus primary accession numbers GSE296200, GSE296201, and GSE296203. The values displayed as individual data points within the quantitative graphs are available in the Supporting Data Values file.

## Author contributions

CJB, DMS, XS, and HS designed and conducted experiments. EFY, NL, YS, and RA provided technical assistance and conducted experiments. CJB, MCK, and CJY performed and interpreted transcriptomic and epigenetic data. BR contributed mice and helpful discussions. JAT and PJT conducted experiments with germ-free and conventionalized mice. CJB, BAM, and AM conceptualized the overall project, acquired funding, supervised experiments, and wrote and edited the manuscript. The order of co–first authors was determined by the volume and conceptual novelty of the work each contributed.

## Supplementary Material

Supplemental data

Supporting data values

## Version 1 09/02/2025

Electronic publication
