# Supplementary material for: A20’s linear ubiquitin–binding motif restrains pathogenic activation of Th17 cells and IL-22–driven enteritis
Source: J Clin Invest. 2025 Sep 2;135(17):e187499. doi: 10.1172/JCI187499 (PMC12404748; doi:10.1172/JCI187499)
Supplement: Supplemental data [file jci-135-187499-s248.pdf]

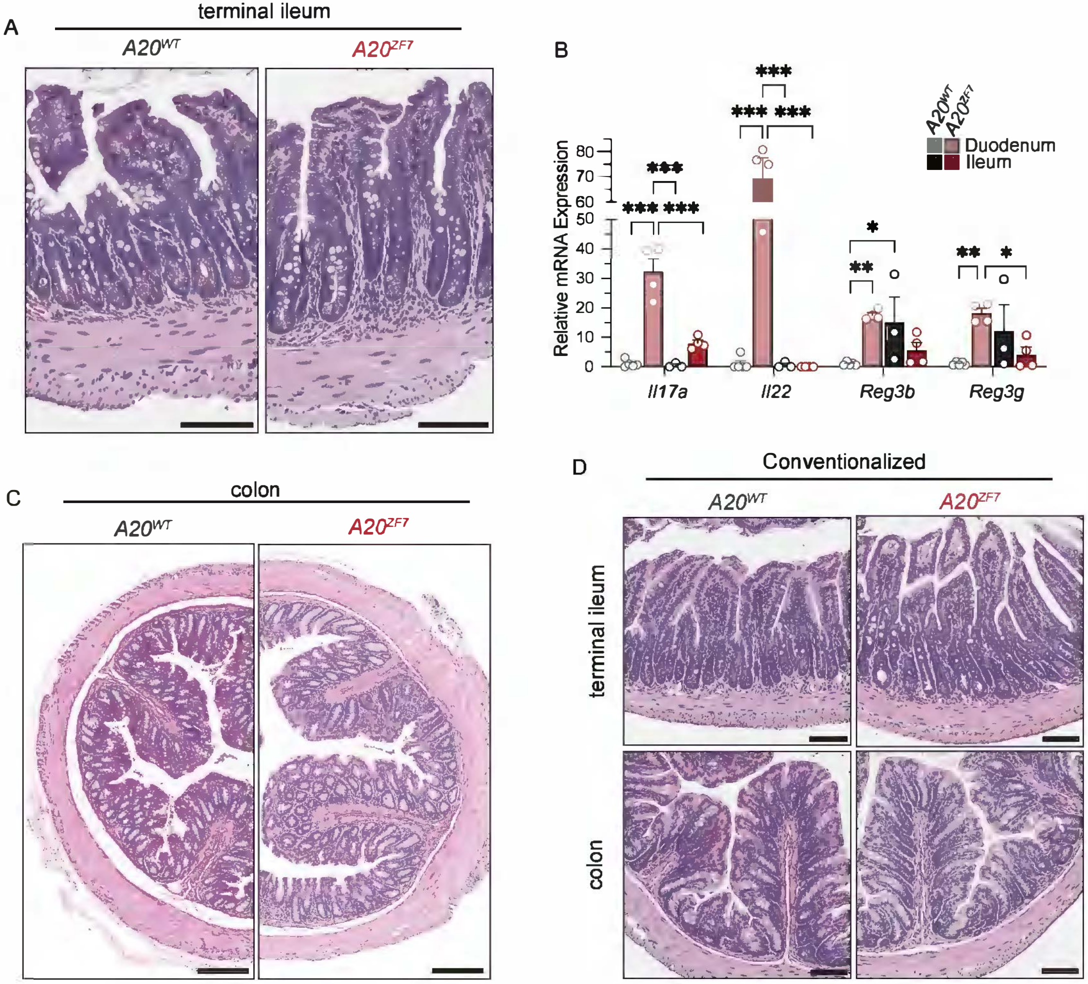

Supplemental Figure 1. Terminal ileum and colons of wild-type and A20^{ZF7} mice show no inflammation.
(A) Terminal ileums from WT or A20^{ZF7} mice. Bar, 100 microns.
(B) qPCR of proximal duodenums and terminal ileums from WT or A20^{ZF7} mice. Two-way ANOVA with post-hoc Tukey multiple comparisons correction with simple effects. *$p<0.05$, ** $p<0.01$, ***$p<0.001$.
(C) Colons from WT or A20^{ZF7} mice. Bar, 250 microns.
(D) Terminal ileums and colons from conventionalized WT or A20^{ZF7} mice. Bar, 100 microns.

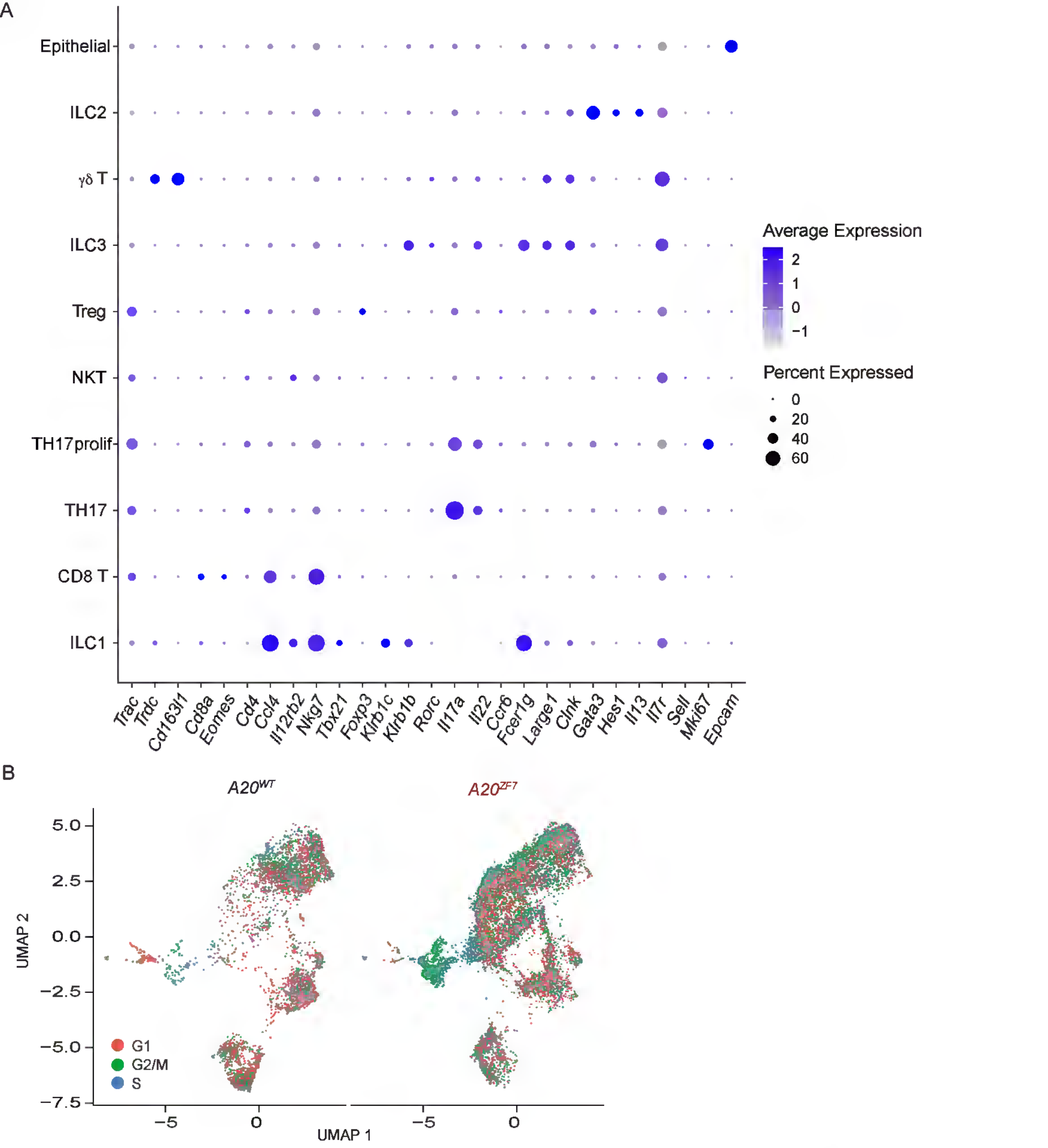

Supplemental Figure 2. Defining cluster identity and proliferative capacity after regression of cell cycle genes.
(**A**) Dot plot of cluster-specific gene expression.
(**B**) Cell cycle scores of WT and A20[ZF7] intestinal lymphocytes.

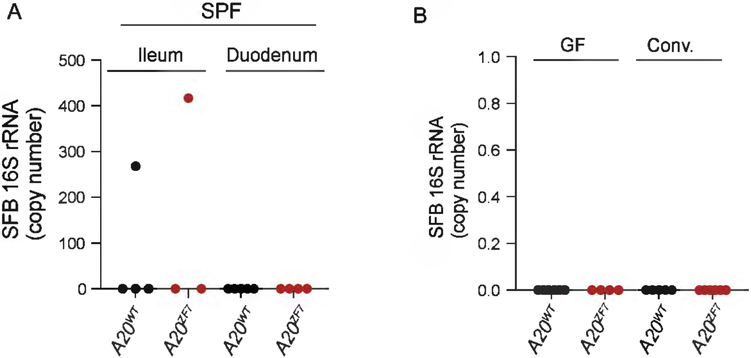

Supplemental Figure 3. SFB is rarely detected in both WT and A20ZF7 terminal ileums but not duodenums. (**A**) SFB 16S rRNA copy number in the ileum and duodenums of specific pathogen-free (SPF) WT and A20^{ZF7} mice. (**B**) SFB 16S rRNA copy number in the duodenums of germ-free (GF) and conventional (conv) WT and A20^{ZF7} mice.

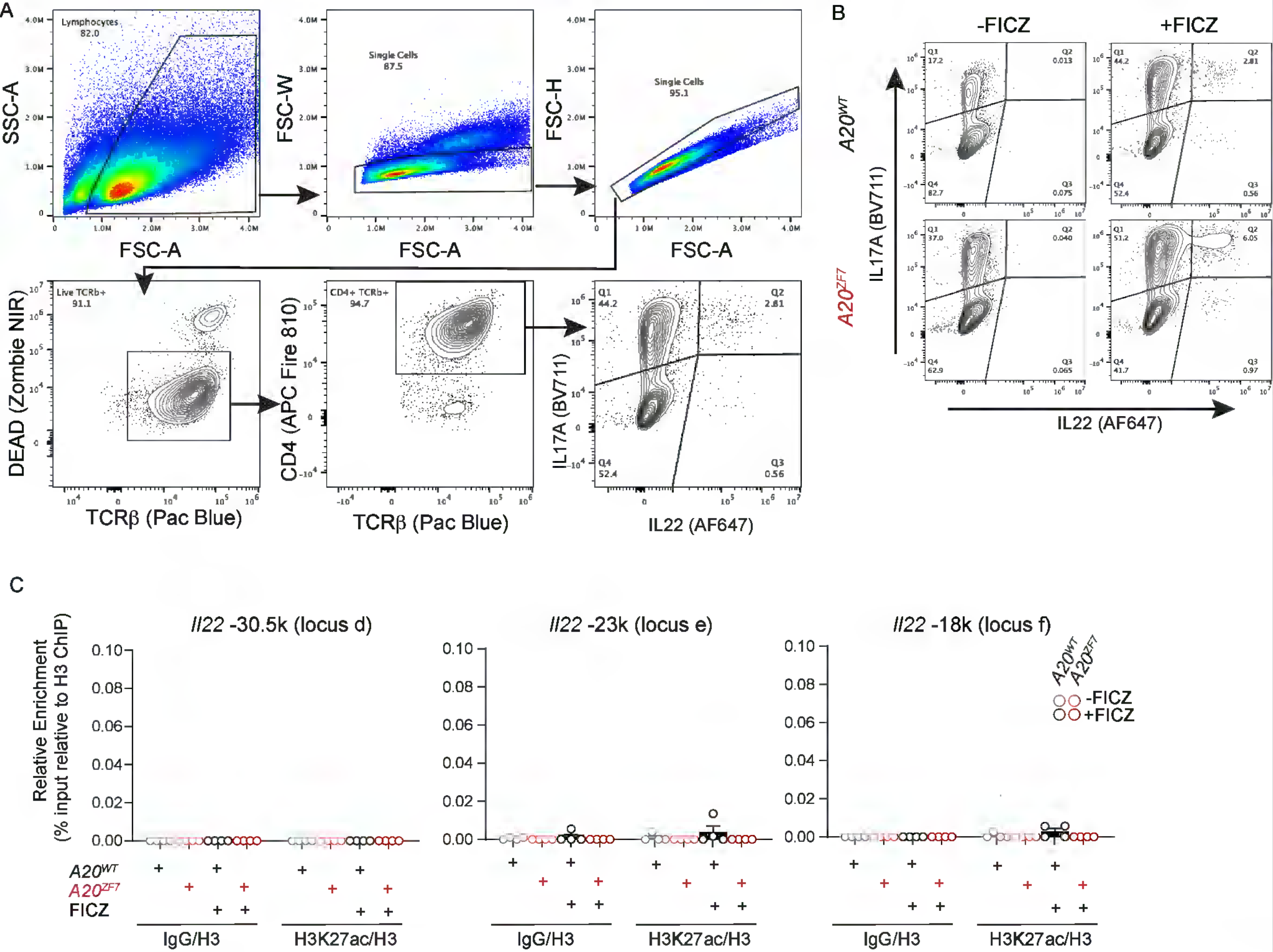

Supplemental Figure 4. IL17A and IL22 expression and acetylation of H3K27 at extragenic ATAC-accessible loci in WT and A20^{ZF7} TH17 cells.

(**A**) Gating strategy to identify live TCRβ+ CD4+ cells expressing IL17A and/or IL22.

(**B**) IL17A and IL22 expression by flow cytometry in murine WT or A20^{ZF7} cells (differentiated in vitro in TH17 conditions in the absence/presence of 100 nM FICZ).

(**C**) Chromatin immunoprecipitation of H3K27ac and irrelevant IgG control at DNA accessible sites.

Data shown as mean + SEM. Statistics calculated using unpaired two-tailed Student t-test with Welch correction.

All comparisons are not significant.

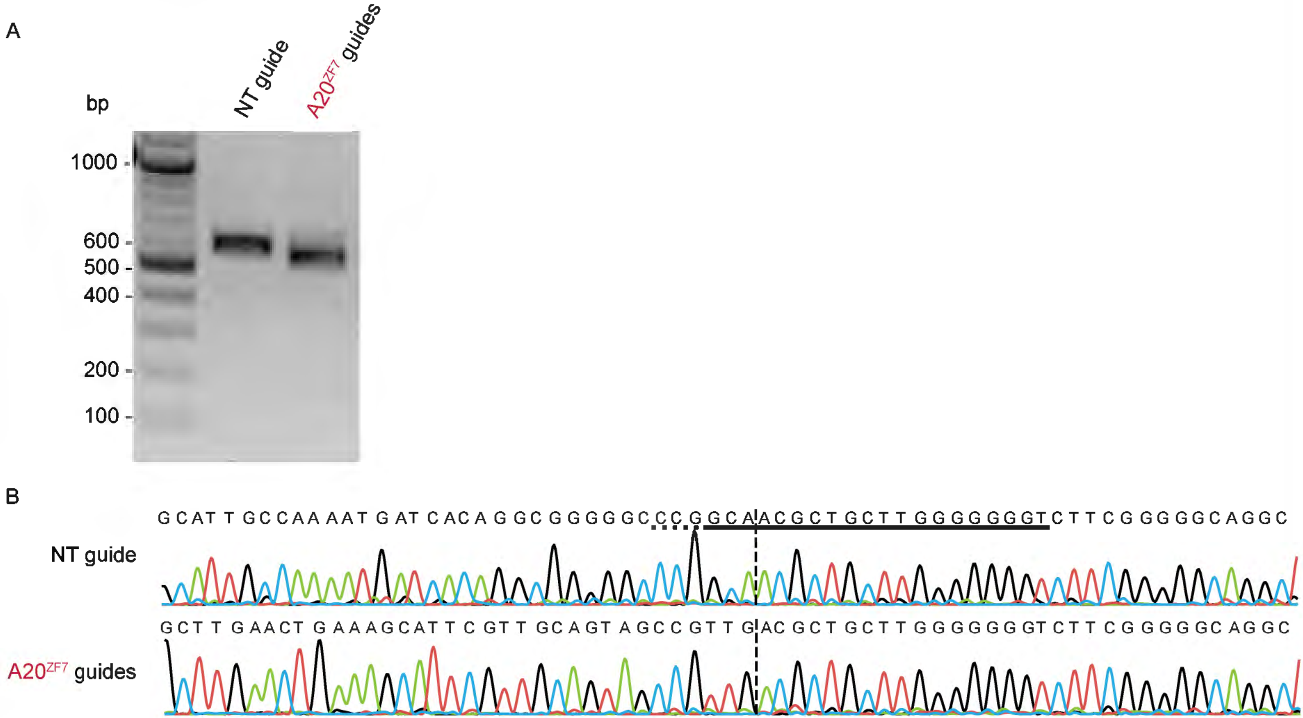

Supplemental Figure 5. CRISPR/Cas9-mediated editing using a dual-guide approach results in highly efficient ablation of A20^{ZF7} domain. Two CRISPR guide RNAs targeting the human A20^{ZF7} domain excise 40 nucleotides from the N-terminal half of the ZF7 domain, excising the first two (of four) zinc-coordinated cysteines and resulting in a frame-shift of the downstream protein-coding sequence.
(**A**) Agarose-TBE gel displaying PCR amplicons of *TNFAIP3*'s ZF7 domain in T cells treated with Cas9 and CRISPR guide RNAs against control/non-targeting (NT) or dual A20^{ZF7} sequences. There is a visible ~40 bp decrease in amplicon size from A20^{ZF7}-ablated cells.
(**B**) Chromatogram of Sanger sequences of amplicons generated in (A) confirm a 40-bp deletion within the ZF7 domain. Underlined nucleotides indicate CRISPR target sequence (black solid line) and CRISPR PAM sequence (red dotted line). Vertical dotted line indicates predicted Cas9 dsDNA nuclease cut site. Synthego's ICE analyses of chromatograms routinely show effective ablation in >85% of alleles.
